# Perceptions and experiences of female nurses when confronted with expressing a conscientious objection towards end-of-life care in Greece

**DOI:** 10.1186/s12912-023-01555-8

**Published:** 2023-10-10

**Authors:** Polychronis Voultsos, Christina-Erato Zymvragou, Nikolaos Raikos

**Affiliations:** Laboratory of Forensic Medicine & Toxicology (Division: Medical law and Ethics), School of Medicine, Faculty of Health Sciences, Aristotle University of Thessaloniki, University Campus, 54124 Thessaloniki, GR Greece

**Keywords:** Nurses / nursing personnel, Conscientious objection, End-of-life care, Futile care, Subservient interactions, Qualitative study, Greece

## Abstract

**Background:**

Conscientious objection in nursing has been a topic of much discussion in recent years. Healthcare providers’ conscientious objection has been included in Greek legislation. However, little is known about the real experiences of nurses who want to apply conscientious objections in their practice. This study aimed to contribute to filling that gap.

**Methods:**

This qualitative study was conducted with eighteen experienced female nurses. Data were collected through semi-structured in-depth qualitative interviews conducted with purposively selected nurses during the period from October 2019 to January 2020. Interviews were transcribed verbatim and analysed thematically. The ethical principles of anonymity, voluntary participation and confidentiality were considered.

**Results:**

Eight major themes and seven subthemes emerged from the thematic data analysis. Oppressive behaviors in the workplace and subservient interactions between nurses and physicians, suboptimal communication and inadequate support of nurses, perceived ineffectiveness of nurses’ conscientious objections, missing legal protection against job insecurity, provision of care labeled ‘futile’, nurses’ false knowledge and perceptions on medical situations related to conscientious objections, nurses’ fears of isolation bullying and negative gossip in the workplace and a trivial amount of nurses’ involvement in medical decisions emerged as barriers to nurses raising conscientious objection. Furthermore, from data analysis, it emerged that some nurses had false knowledge and perceptions on medical situations related to conscientious objections, some nurses experienced mild uncertainty distress about their ethical concerns, nurses considered their remote contribution as participation that can give rise to conscientious objection, a collective conscientious objection raised by nurses might have increased chances of being effective, and upbringing, childhood experiences, education and religion are factors shaping the nurses’ core values.

**Conclusion:**

A total of fifteen themes and subthemes emerged from this study. Most of the findings of this study were previously unknown or undervalued and might be helpful to inform nurses and nursing managers or leaders as well as healthcare policy makers. The results of this study might contribute to addressing the need for creating ethically sensitive health care services and ensuring nurses’ moral integrity and high quality of patient care.

**Supplementary Information:**

The online version contains supplementary material available at 10.1186/s12912-023-01555-8.

## Background

### Conscientious objection in health care settings

Conscientious objection is a complex topic of great clinical and philosophical importance. In the context of contemporary medical ethics, the legitimacy of conscientious objection is an increasingly contentious issue that currently came again under fire, fueling heated debate between scholars. However, it is a difficult task to balance the conflict between patents’ right (based on autonomy and equality of access to healthcare services) to access legal health services and healthcare professionals’ right (based on autonomy and moral integrity) to practice with respect to their own consciences. Importantly, the ever-accelerating advance of medical technology has resulted in an increasing number of newly appearing ethical dilemmas.

On the one hand, a widespread assumption has been accommodated for far too long in the field of medical ethics that healthcare professionals must be allowed the right to conscientious objection. From this perspective, it is argued that healthcare professionals’ deeply held (core) values and personal beliefs deserve respect if a medical procedure goes against these values and personal beliefs, as well as their autonomy and moral integrity. Positions in favor of conscientious objection accommodation are called ‘pro-choice’ positions. In principle, healthcare professionals should be provided adequate room to exercise conscientious objection. However, typically, CO occurs only when health providers ‘refuse to provide legal, professionally accepted, clinically appropriate medical services’ [1]. In that regard, Wicclair defends ‘a more nuanced contextual approach that includes constraints on accommodation’ [1].

The accommodation of conscientious objection aims to secure health providers’ moral integrity and ethical well-being [2]. Moral integrity is a moral, complex and abstract term that means unity between personal and professional values and responsibilities [2].

On the other hand, scholars have taken a clear stance against the legitimacy of conscientious objection in health care, arguing that it goes against the core professional values that a health professional has accepted when voluntarily choosing to join the particular profession [3]. Even proponents of conscientious objection accommodation acknowledge that constraints should be placed on it when conscientious objection is destructive to individuals and society and that not all values and personal beliefs can form a licit base of conscientious objection [4, 5]. Furthermore, patients claim a legitimate right to undergo a treatment that they are entitled to. Fiala and Arthur stated that ‘individuals should not be allowed to boycott a democratically decided law because of society’s deference to religious beliefs and traditional views that assign women to a childbearing role’ [6]. Importantly, CO ‘can be disruptive in a variety of ways and may disadvantage patients’ [7]. Positions against conscientious objection accommodation are called ‘absolutist’ positions. However, it should be noted that neither absolutist nor pro-choice positions are satisfactory. Not surprisingly, there have been an increasing number of scholars in search of a compromise solution between conscientious objectors and patients. Schuklenk puts it best in saying that ‘the legal literature on the subject is growing due to the impossibility of satisfactory compromises’ [8]. In addition, to date, scholarship has not provided a satisfactory answer on what is a blameworthy contribution to principal moral wrongdoing.

The debate over the accommodation of conscientious objection in the context of clinical health care is often confined to physician objectors, thus placing less considerable emphasis on other healthcare providers. Fleming and Frith et al. state that ‘an invisibility of midwives and nurses exists in the whole debate concerning conscientious objection reflecting a gap between literature and practice…While the arguments in the literature emphasize the need for provision of conscientious objection, a balanced debate is necessary in this field, which includes all relevant healthcare professionals’ [9].

The ever-accelerating advance of medical technology has resulted in an increasing number of newly appearing ethical dilemmas being raised. Moreover, in health care settings, there have been changes in the provision of services in various areas of medicine because the legal framework concerning certain medical procedures has been liberalised and, hence, these medical procedures have gained popularity. Given this liberalisation, nurses should accommodate their CO in the new legislative landscape [10]. Therefore, an increasing number of healthcare professionals may find themselves troubled by questions of conscience over newly appeared ethical issues and dilemmas.

Although many authors have addressed this issue in the literature, there is a lack of empirical research on the topic [11, 12]. Nurses act in very complex and pluralistic contexts [13, 14]. Lamb states that it is difficult to make sense of conscience in the context of health care [2]. She puts it best in saying that conscience is ‘largely absent in definition or in common definition in the discourse surrounding conscientious objection in health care practice’ [2]. Jodaki et al. concluded ‘that conscience is an inner feeling or voice’ that ‘testifies to the rightness or wrongness of an action’ and ‘plays a vital role in providing ethical care by nurses’ [15]. In that regard, Jodaki et al. concluded that ‘nurses expressed the importance of following the call of conscience at their workplace, which demanded unlimited efforts to achieve a clear conscience’ [12].

At any rate, further research is needed to understand how nurses respond to conscience concerns in morally pluralistic nursing contexts [16]. This understanding is fundamental to advancing ethical nursing practice. The meaning of conscience for nurses in the context of CO is not clear and needs to be further explored [17]. The exploration of the concept of conscience in the context of conscientious objection is relevant to fully understand ethical nursing practice [17]. Not surprisingly, Lamb, Babenko-Mould et al. remark that ‘addressing ethical issues in nursing practice is complex’ [18]. Then, they add: ‘… addressing challenging ethical questions as well as the concept of conscience are relevant to advancing nursing ethics and ethical nursing practice’ [18].

While conscientious objection in the context of healthcare is one of the most controversial topics in bioethics, few empirical studies have explored nurses’ conscientious objection. In Greece, there is a lack of empirical evidence to support an understanding of what it is like for nurses to make a conscientious objection. In addition, Greece is a country to be explored with regard to this topic for the following reasons: the Greek Orthodox Church may profoundly shape the population’s beliefs and values, which may form a ground of conscientious objection to medical procedures such as induced abortions, end-of-life decisions and assisted fecundation and reproduction. Moreover, Greece is a country that belongs to the so-called ‘Mediterranean ethics’ zone of Southern Europe. The so-called ‘Mediterranean ethics’ has its roots in ancient Greece (Ippokrates, Plato and Aristotle) and Christianity (along with Islam and Judaism, especially in regard to other countries of the Mediterranean Basin). Among others, it is characterized by considering patients from a societal perspective and a pro-life consideration [19].

## Conscientious objection in nursing: the greek normative framework

The inherent moral nature of nursing is indisputable. Nurses spend a considerable amount of time providing hands-on care for patients. Conscientious objection in nursing is an ethical concept to support nursing practitioners in addressing issues of conscience amid ethically challenging situations. More precisely, conscientious objection in nursing is nurses’ refusal to perform an action (e.g., provide care) or participate in an activity they consider to be morally or ethically wrong and contravene their core values and personal beliefs. Conscientious objection in nursing is a topic of much and ongoing discussion in recent years [20, 21]. In fact, it is essential for professional ethics to facilitate nurses to act as moral agents in accordance with their core values, personal beliefs and conscience. As the roles of nurses are expanded in the modern healthcare context, ethical dilemmas in nursing practice are becoming increasingly common [20]. Conscientious objection is intrinsic in clinical nursing practice and an important part of it [20]. However, ‘little is known about real experiences of nurses who apply objections in their practice’ [20]. While conscientious objection is a well-known phenomenon in the literature, there is not adequate evidence on what is like for nurses to have ethical concerns and make conscientious objection in clinical practice. Empirical research from across Europe has argued strongly for and against conscientious objection in nursing, with the arguments against it being mostly related ‘to organizational aspects of its application’ [20]. In that regard, Czekajewska et al. recently stated that while in Polish society there is an ongoing heated debate on nurses’ right to invoke their conscientious objection, little is known about their attitudes towards that matter [22]. Much of the same holds for Greece, where little is known about the perceptions and lived experiences of nurses who have ethical concerns and conscientious objections in clinical practice. In that connection, it is to be examined to what extent nurses can opt out of clinical procedures on the basis of conscience under the current normative framework. Conscientious objection has been included in Greek legislation as a safeguard to protect the rights and moral integrity of healthcare professionals. Currently, however, as in other countries, there is no clear normative framework with regard to this particular issue. In Greece, physicians are allowed to claim the status of conscientious objector under law 3418/2005, namely, the Code of Medical Deontology (article 2§ 5). Regarding nurses, the legal landscape is less clear. Article 20 of the Code of Nursing Care Deontology (Presidential Decree No 216/2001) states that a nurse can opt out procedures of abortion or assisted reproduction when this goes against their deeply held convictions. It does not make reference to other areas of health care. In addition, the Code of Nursing Care Deontology in many articles implicates the nurses’ primary obligation to respect the patient’s care as care provided for a bio-psycho-social and mental being and the patient’s dignity, personality, autonomy, freedom, and best interest as well. It calls for promoting human life while being silent with regard to euthanasia. Note, however, that the aforementioned Code in article 13 explicitly recognizes the scientific independence of a nurse who, importantly, views as equal member of the therapeutic team. Last, it is noticeable that the Greek Code of Nursing Care Deontology in article 13 states that nurses should adhere strongly to physicians’ proper orders. This statute might be interpreted against the accommodation of nurses’ right to conscientious objection.

In short, the Greek normative framework concerning nurses’ right to conscientious objection is less than perfect. This fact places them at an unfair advantage in comparison with physicians. Moreover, empirical research on the topic of conscientious objection among nurses is lacking. Given that situations involving end-of-life patients are extremely challenging from an ethical viewpoint, most nurses’ ethical concerns are related to end-of-life care. The present study aimed to contribute to filling this gap in the context of end-of-life health care. The authors (researchers) choose to focus their research on only female nurses, given that there may be gender differences in nurses’ perceptions of and experiences with conscientious objection. Literature argues that female nurses tend to display higher levels of moral sensitivity regarding the terminally ill compared to male nurses [23]. In that regard, O’Connell conducted a study and found that in critical care nurses ‘females reported statistically significantly higher moral distress scores than did males’ [24]. Male nurses’ perceptions of and experiences with conscientious objection would be a different topic of interest.

## Research objectives

Primary research objective:

- To gain a deeper insight into nurses’ perceptions and experiences of making conscientious objection to participating in nursing activities to be executed in situations involving end-of-life patients.

Secondary research objective.

b) To illustrate unknown or undervalued complexities of nurses’ issues of conscience amid ethically challenging end-of-life situations.
c) To identify barriers and facilitators of addressing nurses’ issues of conscience amid ethically challenging end-of-life situations.

## Methods

### Study design

The present study was a qualitative research study based on in-depth interviews conducted with 18 experienced frontline nurses who were currently working and previously had been working for at least ten years as frontline nurses on hospital wards that have beds for terminally ill patients. This qualitative descriptive study was conducted from October 2019 to January 2020. Thematic analysis was selected as the methodological orientation of the study.

### Participants and setting

#### Participants’ recruitment

The researchers used a purposive sampling method. Participants were approached through researchers’ personal acquaintances. Furthermore, the researchers strived to enhance the validity of the study by using maximum variance in participant selection by considering the ward in which participants were working at the time of the interviews. The participants in this study came from various departments and wards of two large tertiary Greek hospitals, covering a wide range of medical areas (apart from the wards presented below). Moreover, all participants said that previously they had been working in various hospital departments covering various medical areas (apart from the wards presented below).

#### Participant characteristics

The eighteen respondents of this study were all women; they were diverse in terms of age, years of worked nursing experience and settings where they were working. Note, however, that the participants in the present study had previously been operating in various hospital wards, apart from dermatology clinics, ophthalmology wards, psychiatric wards, pediatric wards, neonatal care units, obstetrics and gynecology wards or facilities that offer infertility services. All participants were nurses with many years of professional experience: Fourteen out of the eighteen participants had more than 15 years of worked nursing experience. None of them had less than 10 years of worked nursing experience as frontline nurses on hospital wards that had beds for terminally ill patients. The mean worked nursing experience of the participants was 18.61 years. The age range of the participants was from 32 to 52 years. Only three participants were younger than 40 years. The mean age of the participants was 43,55 years. At the time of the interviews, all participants were employed by the Greek National Health Service and were working on various wards in two large tertiary university hospitals (named ‘Hippokrateion’ and ‘AHEPA’, respectively) in Thessaloniki (the second largest city in Greece). All participants were graduate degree holders. Participants were given information explaining the research and were assured of confidentiality; to preserve their anonymity, numbers (i.e. N1) are used in this paper.

#### Inclusion criteria

Female nurses must have met all of the following inclusion criteria to be eligible for participation in this study: (a) being currently working as frontline nurses on hospital wards that have beds for terminally ill adult patients and (b) having been working as frontline nurses on such wards for at least ten years.

#### Exclusion criteria

Female nurses who (a) did not understand the purpose of the study, (b) were not interested in participating in the study, or (c) were not enthusiastic about participating in the study were excluded.

#### Setting

All interviews were carried out face-to-face at the participant’s workplace, with nobody else present, and lasted approximately 40 min to an hour. They held in comfortable, neutral and quiet places to ensure privacy and confidentiality while minimizing the environmental impact.

### Procedure

#### Interviews

Data were collected from October 2019 to January 2020 through semistructured in-depth interviews conducted in person. Data collection ceased only when data saturation was reached. Thus, data collection continued through 15 interviews. Three additional interviews were conducted to ensure data saturation. All interviews were carried out by a female researcher (C-EZ) trained in qualitative research interview techniques. Field notes were taken immediately after each interview and were used to inform the researchers who conducted data analysis. The researcher observed the facial expressions and body language of participants, which may be useful for the analysis. Data were gathered by combining conversational interviewing and structured question interviewing to help produce a comprehensive set of insightful findings. The participants did not provide feedback on the findings.

#### Interviewer-interviewee relationship

To ensure trustworthiness in the study, the interviewer spent time beforehand to gain participants’ trust and make them feel comfortable. She had previously explored her own perspective and was emotionally prepared to be able to control her possible influence on the interview. She presented herself as having no strong moral views on the issue of conscientious objection. She made an effort not to ask leading questions. She did not interrupt the participant while they were speaking or remaining silent. As a phenomenological researcher, she maintained an unreflective and normal attitude to prevent unintentional personal bias. Reflexive thinking was employed throughout the research process to reduce unwitting personal bias.

#### Interview guide

The interview guide was developed based on the available literature. It was slightly refined after the initial results from a few interviews to become more suitable for the purpose of obtaining a better understanding of the specific issues being questioned. The interview guide used in this study centered on exploring the lived experiences of nurses who at the time of the interview were working on various wards in two great tertiary hospitals in Greece. The participants were encouraged to expand upon topics such as ‘caring for critically ill patients’ or ‘being involved in end-of-life decisions.’ They were asked to expand on their perceptions and experiences of their conscientious objection to providing care for end-of-life patients.

The interviews were semistructured and started with questions such as ‘Can you please describe in detail your experiences of dealing with ethically challenging situations involving end-of-life patients?’ (*a grand tour question to make the participant comfortable*), ‘Sometimes, nurses may feel reluctant to provide care or participate in an activity they consider to be morally wrong and contravene their core values and personal beliefs. Can you please describe in detail what is it like for you (as a nurse) to have ethical concerns about participation in caring for end-of-life patients or involvement in end-of-life decisions?’, ‘Can you please describe in detail your experiences (if any) of raising ethical concerns or conscience-based objection to participate in nursing activities to be executed in situations involving end-of-life patients?’ ‘Can you please describe in detail your lived experiences (if any) of seeing other nurses raising conscience-based objection to participate in nursing activities to be executed in situations involving end-of-life patients?’ or ‘Can you please describe in detail what challenges you faced (if any) when attempting to openly express your conscience-based objection in your hospital organization? ‘.

Additional questions were asked to elicit more detailed explanations and to identify the essential themes of nursing practitioners’ perceptions of the topic of interest.

### Data analysis

Thematic analysis was selected as the methodological orientation of the study and used to analyze the qualitative interview data [25]. In the modern research landscape thematic analysis continues to be considered a ‘powerful and flexible method of qualitative analysis’ [26]. Interviews were audio-recorded, manually transcribed verbatim, thematically categorized, and analyzed. In addition, field notes were used for recording nonverbal behavior patterns. Given that all interviews were carried out face-to-face, non-verbal communication cues such as the whole-body language or emotional responses were observable [27]. The contextual information that was obtained was useful to improve and deepen the data analysis.

In the first step (familiarization with the data), the researchers went through the raw data by reading and reviewing transcripts independently and repeatedly to gain a deeper understanding of them. In the second step (generating initial codes), data-driven themes were identified. The authors created initial codes from the interview data and categorized them thematically. In the third step (searching for themes), the transcripts were annotated with numerical codes according to the themes identified. Initial codes were going to form a set of candidate themes and subthemes. In the fourth step (reviewing themes), the authors considered whether the formed candidate themes might form real themes. The authors assessed whether these themes ‘work’ in relation to the data set [25]. In the fifth step (defining and naming themes), the authors conducted a detailed analysis for every single theme. The authors examined ‘how it fits into the broader overall „story‟ that you are telling about your data, in relation to your research question or questions’ [25]. Furthermore, the authors examined the relationships and interactions of the identified themes. At the end of this step, clear and distinct themes were identified.

Additionally, NVivo software was used to secure the systematic character of data analysis. The entire coding process was aided using computer-assisted qualitative data analysis software (CAQDAS) (NVivo, 2015). Furthermore, during the translation of the written narratives (quotations) into English, special care was taken to ensure that the meaning of the original texts remained unaltered in this final paper.

### Techniques to foster rigor and trustworthiness

The researchers worked in accordance with the four dimensions criteria methodology described by Lincoln and Gupta with the aim of ensuring rigor (including validity and reliability) and trustworthiness in this study [28]. Credibility, transferability, dependability and confirmability were the four key criteria to be considered [28]. The authors used the techniques of ‘peer debriefing’ and ‘investigator triangulation’ to foster both credibility and confirmability in the research. The first author (PV) and another nurse researcher (AT) served as peer debriefers to examine the findings. AT is a PhD candidate with experience in conducting qualitative research with nurses. Furthermore, a discussion was conducted to balance out the researchers’ interpretation biases (intentional or unintentional). To foster trustworthiness (credibility), field notes from each interview were recorded to maintain a systematic audit trait. Moreover, the researchers applied the reflexivity technique (including reflexive dialogue) in data analysis to enable increased confirmability in the research. All researchers carried out an in-depth reflection and engaged in a creative conflict process (interactive introspection) with the aim of exploring (and being aware of) their own internal assumptions, values, and biases or failures. In addition, to ensure transferability of the findings the authors strove to create an Interview Guide that would encourage participants to describe the phenomenon of interest in sufficient detail. Sufficient quantity and quality of data (thick-rich description) should be collected before data saturation has been reached [29]. Furthermore, purposive sampling (involving source triangulation) and theoretical saturation were used to foster the potential for transferability in the research. Iterative sampling was achieved through regular meetings until no new codes emerged from the research data. The progress of the research was monitored by the research team on a weekly basis.

For more information in respect of the techniques used by the interviewer to foster the trustworthiness in the research see the abovementioned ‘Interviewer-interviewee relationship’ subsection.

## Results

Among other findings, from data analysis emerged that a number of organizational and cultural barriers were identified to hamper participants’ ability to express a conscience-based objection towards providing end-of-life care. However, all participants believed that a nurse should be allowed to decline their participation in medical procedures, which, in their opinion, goes against their core values and deeply held beliefs.

Eight major themes and seven subthemes emerged from the thematic data analysis (Table 1).

**Table 1:** Major themes and subthemes

Theme	Subtheme
1.1. Oppressive behaviors and interactions in the workplace emerged as barriers to nurses raising conscientious objection.	1.2. Subservient interactions in the workplace emerged as barriers to nurses raising conscientious objection. 1.3. Perceived ineffectiveness of conscientious objections emerged as a barrier to nurses raising them. 1.4. Fears of isolation bullying and negative gossip in the workplace emerged as barriers to nurses raising conscientious objection.
2. Suboptimal communication and inadequate support at work emerged as barriers to nurses raising conscientious objection.	2.1. Trivial amount of nurses’ involvement in medical decisions emerged as a barrier to nurses raising conscientious objection. 2.2. Nurses believe that collective conscientious objection raised by nurses might have increased chances of being effective.
3. Missing legal protection against job insecurity emerged as a barrier to nurses raising conscientious objection.	
4. ‘Futile care’ emerged as main reason behind conscientious objection.	4.1. Most nurses adopted a strong stance (for different reasons) against providing the so-called ‘futile care’. 4.2. A few nurses adopted a strong stance (for different reasons) against avoiding or stopping the provision of the so-called ‘futile care’.
5. Nurses experienced mild uncertainty distress about their ethical concerns.	
6. Some nurses had false knowledge and perceptions on medical situations related to conscientious objections.	
7. Upbringing, childhood experiences, education and religion emerged as factors shaping the nurses’ core values.	
8. Nurses considered their remote contribution as participation that can give rise to conscientious objection.	

### Oppressive behaviors and interactions in the workplace emerged as barriers to nurses raising conscientious objection

#### Subservient interactions in the workplace emerged as barriers to nurses raising conscientious objection

A theme that emerged from the data analysis was that physician oppressive behaviors and attitudes toward nurses often reinforce historical hierarchies of power and hold nurses back from reaching their full potential in expressing their ethical concerns. The majority of the participants in the present study (eleven out of a total of eighteen participants) explicitly supported the idea that subservient interaction, namely, power imbalance between physicians (or nursing managers) and nurses, was a substantial factor constraining them to abstain from raising conscientious objection. This was the most recurring finding in data analysis. Participants felt that this may arise from *unequal power dynamics* between physicians and nurses. They felt hampered from expressing their conscience-based refusal and forced to act in conformity with the physician recommendations and participate unwillingly.

Furthermore, almost half of the participants (eight out of a total of eighteen participants) had a minimalist, defensive and rather vague perception of their own professional role under the current legal framework. Participants appeared to be convinced (to a greater or lesser extent) that their current role as nurses did not allow them to have a say in decisions concerning end-of-life care for their patients. As presented below, a trivial amount of nurses’ involvement in medical decisions emerged as barriers to nurses raising conscientious objection. Participants perceived their professional role as merely executive agents who were acting on physicians’ behalf. From the interview data of this study, the authors built the picture of nurses who were expected to adopt a role that is subservient to physicians. The assumption of physicians having exclusive right to make moral judgments on medical treatments was also common among the participants. Participants regarded themselves as having the moral and professional obligation to act in conformity with the physician recommendations. Some participants seemed to believe that this was a justified consideration. Reading between the lines, the researchers would say that in all likelihood participants had internalized the exercise of physician authority as a type of environmental oppression that is inherent to their profession. Participants N13 and N16 seemed to have internalized the subservient interactions in their hospital organizations.

Participant N13 declared that she is a nurse and therefore she could not have conscientious objections, at least under the current regulatory framework. She said,

…having objections…I strongly believe it is unethical to bring such objections on working issues. saying “No” is hard… transfusion for a patient at the end-of-life stage… there, I do have objections but I must proceed if I am asked to do so… there is always a dispute among nurses and doctors, especially on taking responsibility. I think that there should be limits.

In a similar vein, Participant N16 said,

…I would do if my supervisor or the doctor would ask me to do so, I wouldn’t do it to dodge any responsibilities….

One participant (N15) was keeping her capability of making moral judgments never cultivated. This was probably due to conformity-based ‘pro-profession’ impulses that might cause nurses to believe that ‘doing so is the norm’. Participant N15 admitted:

I could have gained more knowledge on this issue, someone could get me alerted…In general I am a “Yes to all” person… I am a very convenient person and my reactions are limited.

Many participants (N1,N2,N5,N6,N14,N16,N17) detailed the subservient interactions between physicians and nurses. Their interview quotes are presented in Supplementary file 1.

#### Perceived ineffectiveness of conscientious objections emerged as a barrier to nurses raising them

Participants seemed to believe that in the case of making conscientious objection, it is most likely that it would be rendered ineffective because of organizational and cultural barriers within the healthcare organization. This belief seemed to operate as a substantial barrier to expressing their conscientious objection. Four participants (N1,N8,N14,N17) considered that on a presumed case of raising objection, they would do it in vain. Based on their previous experiences, they believed that their voice would be lost. They were of the belief that in case of making conscientious objection, the denied contribution to a medical procedure would be performed by another health professional. Participants’ interview quotes are presented in Supplementary file 1.

#### Fears of isolation bullying and negative gossip in the workplace emerged as barriers to nurses raising conscientious objection

Reading between the lines, the researchers would say that participants’ fears of isolation bullying and negative gossip in the workplace emerged as barriers to nurses making conscientious objections. This finding came up repeatedly in data analysis. Participants offered insight into how gossip or criticism at work might be highly influential in creating barriers to nurses raising conscientious objection. Participants feared that in case of raising moral concerns, other healthcare providers (coworkers) would mock or disdain them, thus causing them to feel isolation bullying and/or perceive negative gossip or even rejection in the workplace, which might be spreading by both physicians and colleagues (chiefs or subordinates). Therefore, in the case of raising conscientious objection, they would view their coworkers with suspicion. The following interview quote is representative to illustrate this point.

Participant N16 said,

*‘There is no suitable system or framework where you can express your opinion and this opinion to be heard without judgment… it is kinda a grey zone for this… only doctors may do so…’*.

Participant N1 explained how she might interact with other healthcare professionals in case of a conscientious objection raised by her. She said,

They may make fun of me or disagree with me… I’d be afraid of their rejection… or being the minority apart from the majority… they may consider I am too romantic and delicate or maybe the doctor may suppose that it is not me who makes the call, only him/her, thus you as a nurse don’t have the right to have a different opinion on certain tactics.

Importantly, some participants placed considerable emphasis on rejection from their colleagues.

Participant N18 said that in the case of raising conscientious objection, she expected to receive rejection:

…only from my supervisor [nurse leader], not the doctor….

To this effect, Participant N1 said,

I think my colleagues would be the most difficult part.

Similarly, Participant N4 said,

…maybe bad reviews for supervisors or the existing nurses… yes… I believe this is it….

Participants with long nursing experience (N5,N6,N8) reported that they had dared say their opinion, though informally. This has mostly occurred between peers, especially in the ICU that ‘*is a separate area where anything may happen’* (N5). Participants’ interview quotes are presented in Supplementary file 1.

### Suboptimal communication and inadequate support at work emerged as barriers to nurses raising conscientious objection

Many participants in this study placed considerable emphasis on optimal communication and support in the workplace and reported these factors as facilitators of making conscientious objections. They highlighted the need for better communication, support, dialogue, information and cooperation in their workplace. Participants seemed to not feel supported in making conscientious objections by their respective hospital organizations. This finding came up repeatedly in data analysis. The following interview quote is representative to illustrate this point.

Participant N11 identified the different values and attitudes between healthcare providers in healthcare workplaces as barriers to communication between nurses and other health providers, which in turn served as barriers to expressing ethical concerns and conscientious objections. She said,

…there might be occasions at work where we must decline but some things are mandatory and they need to be done… it has to be done… we need to follow the doctor’s orders… it is a value matter, the people you will work with and the mentality… I do not have the same values with you….

Participant N6 was in a similar vein. Her interview quote is presented in Supplementary File 1.

Moreover, some Participants (N9,N11,N13) said that they needed more support in her workplace to feel able to openly raise conscientious objection. Participants’ interview quotes are presented in Supplementary file 1.

#### Trivial amount of nurses’ involvement in medical decisions emerged as a barrier to nurses raising conscientious objection

A trivial amount of nurses’ involvement in medical decisions emerged as barriers to nurses raising conscientious objection. Nurses should be substantially involved in the team in terms of having a say in making decisions about medical treatments. Participants highlighted the need for nurses’ participation in shared decision-making processes. The following interview quote is representative to illustrate this point.

Participant N6 highlighted what is like for nurses to participate in shared decision-making processes. She said,

to start…let us say if there was more discussion on certain issues… if I could participate to the procedure where decisions are taken, I don’t mean that I should be the one deciding, because of course there are people above us, like doctors and they should be the ones who take all decisions, I only imply that I could participate… This only is a huge thing for us nurses….

Participant N13 was in a similar vein. Her interview quote is presented in Supplementary File 1.

#### Nurses believe that collective conscientious objection raised by nurses might have increased chances of being effective

Two participants in this study supported the idea that a collective conscientious objection raised by nurses might have increased chances of being effective compared with an individual conscientious objection.

Participant N18 said,

…all together… maybe this way because the other way that I tried it on my own I had no support… and if I did have any this would be from my supervisor.

Participant N14 was in a similar vein. Participant’s interview quote is presented in Supplementary File 1.

### Missing legal protection against job insecurity emerged as a barrier to nurses raising conscientious objection

All participants complained of the state’s failure to have enacted a law implementing the nurses’ right to conscientious objection without the nurses’ fear of job loss. They complained of a lack of legal security pertaining to their professional rights related to conscientious objection. All participants implied that a lack of clear guidance for their clinical practice was a substantial barrier to expressing their conscientious objection. All participants in this study identified the lack of legal clarity and protection against job loss (especially in the private healthcare sector) as a substantial factor giving rise to feelings of job insecurity. While participants of this study initially defended their conscientious objection as fair and reasonable, during the flow of the interviews, they showed noticeable anxiety and fear of job insecurity. They felt threatened by job loss, namely, dismissal from their job, especially those having been working in the private health sector.

All participants in the present study wished for and recommended legal clarity on their professional rights to object on conscientious grounds. In addition, they felt that it was imperative that lawmakers provide an appropriate legal framework for the purpose of improving their professional dignity and role in terms of having a say in making decisions about medical treatments without fear of unemployment, especially when the conscientious objectors are private sector employees.

Representative interview quotes (from participants N1,N3,N6,N7,N8,N11,N18) are presented in Supplementary file 1.

### ‘Futile care’ emerged as the main reason behind conscientious objection

The so-called ‘futile care’, namely, the provision of medical treatment that uselessly prolongs the life of a terminally ill patient, was a procedure that the majority of the participants in this study regarded as giving rise to ethical concerns and conscientious objection. This was a recurring finding in data analysis.

Most participants were against providing care that they considered ‘futile’, with some participants being in favor of providing care that others considered ‘futile’. Participants showed different reasons behind their attitudes towards providing or not providing ‘futile care’. This reflects the diversity of their moral values.

#### Most nurses adopted a strong stance (for different reasons) against providing so-called ‘futile care’

Most participants took a strong stance against providing futile care based on their values and previous professional experience. The following quotes are representative to illustrate this point.

Participant N3 and Participant N9 expressed definite concerns about providing ‘futile care’.

Participant N3 said,

in terms of transfusions, when this occurs to last stage cancer patients, and they only have hours of living. and they [physicians] perform blood transfusions for them… there was a case that the patient died while we were tried to transfuse and the bottle just went to waste.

Similarly, Participant N9 said,

my only issue is that when you realize that the patient has only hours to live and we keep on giving them antibiotics, and insist on chemo, blood transfusions etc.

Participant Ν16 reported that she was faced with a difficult dilemma. Participant N5 described in detail her previous experience. Participants’ interview quotes are presented in Supplementary file 1.

#### A few nurses adopted a strong stance (for different reasons) against avoiding or stopping the provision of so-called ‘futile care’

Participants N7, N8 and N12 were against stopping providing treatment that others consider ‘futile care’.

Participant N8 was against withdrawing and withholding treatments as ethically different. She said in an emphatic manner,

…you can’t once you intubate a human being go back and say ok that’s it. I’m done… there may not be a change to the patient’s situation but morally I can’t turn off the machine just because someone says so.

Surprisingly, Participants N7 and N12 said that they did not hesitate to act on her conscientious objection to stop providing ‘futile’ care. They appeared to have great respect for human life. Participants’ interview quotes are presented in Supplementary file 1.

One participant (N1) took a strong stance in favor of providing care that others label ‘futile’ because she attributed great importance to the final stages of a terminal illness and the end-of-life period. She placed considerable emphasis on the value of communication between patient and loved ones during this period of time. She said she *‘consider this time period extremely important.’* Most interestingly, Participant N1 stated explicitly that her attitude was based on her empathy-driven emotions and her previous personal experiences. She described her experience of giving end-of-life care for her mother. Participant’s interview quotes are presented in Supplementary file 1.

### Nurses experienced mild uncertainty distress about their ethical concerns

While most participants appeared to be convinced about the correctness of their ethical concerns, a few (two) participants appeared to have some reservations about the correctness of their ethical concerns. They were aware of their ignorance and desired to be able to reflect on whether to participate in a nursing activity or not.

In that regard, it is to be noted that while participants were detailing their lived experiences related to their participation in providing life-sustaining end-of-life treatments, the authors of this study read between the lines (and with help of field notes) that participants had a strong sense of moral responsibility towards patients.

Participant N16 defended nurses’ right to make conscientious objections but had some reservations about the correctness of their ethical concerns. She said,

I believe that we all make mistakes, we do have professional knowledge; thus, we may express our opinion even if that is wrong, if it is it up to the other colleagues to convince us why it is wrong by using legitimate arguments.

Furthermore, Participant N11 highlighted the nurses’ need to reflect upon the correctness of their ethical concerns and said,

up to the extent where we may say that there is no more to be done, the patient is at his/her home. Meaning that we need to think about the decisions…things are not so simple as we imagine them to be….

### Some nurses had false knowledge and perceptions of medical situations related to conscientious objections

Some participants had false perceptions of medical issues related to conscientious objection in nursing, most likely because of inadequate knowledge about end-of-life issues. Two participants wished there were legal provisions for setting a clear cut-off point at which the duty of providing life-prolonging treatment to a terminally ill patient is no longer applicable (Participants N5 and N12). Furthermore, one participant (N13) said that she would not hesitate to decline to provide nutrition support to terminally ill patients on her (arbitrary) perception that is harmful to patients. It is most likely that these participants were unaware of the intrinsic uncertainty involved in a moral judgment about what is called ‘futile care’. Participants’ interview quotes are presented in Supplementary file 1.

### Upbringing, childhood experiences, education and religion emerged as factors shaping nurses’ core values

The quality of the education they had received, the way they were raised in their family and religion emerged as the most important factors shaping participants’ core values on which a conscientious objection might be grounded. Upbringing and childhood experiences came up more repeatedly than education and religion.

Participant N11 stated, *‘the family, they way we were brought up. all these things… the experiences… from childhood.’*

Furthermore, Participant N10 said, *‘…the way I grew up.’*

### Nurses considered their remote contribution as participation that can give rise to conscientious objection

Nurses considered their remote contribution (i.e. preparing a tray) to a medical procedure that they considered ethically wrongful as participation that can give rise to conscientious objection.

Participant N14 said,

…*I kinda agree [with raising objection] … from the moment that I’ll prepare it.*

In the same vein, participant N1 said,

…they could easily let me know I would be sacked in case I didn’t prepare the tray as asked.

One participant said that she would only get involved to the extent that she would not feel morally guilty at participating in such a procedure. Nurse N4 said,

I would only get involved to the extent that I’m ok with that.

## Discussion

### Subservient interactions and bullying in the workplace

Subservient interactions between physicians (or even nursing managers) and nurses emerged as substantial barriers to nurses raising effective conscientious objection. This is not surprising because nursing personnel in Greece are not treated by physicians as professionals who are colleagues on shared work projects.

While nurse-physician collaborative relationships are traditionally characterized by subservient interactions, namely, nurse subservience and physician dominance, in the contemporary context of nursing schools, it is emphasized that physicians and nursing personnel should collaborate as colleagues [30]. This is in line with the fact that in many countries, there is university-level education in nursing. At any rate, the role that the existing health-system framework assigns to nurses is of great importance. It should be highlighted that the structure of the modern health care system has replaced the traditional individual physician-healer by a healing team consisting of various health providers. Nurses play a critical role in curative and preventive care. They are necessary to meet the goals of population health and patient satisfaction. The vast majority of health care services are mediated through nurses.

Importantly, until a few years ago in Greece, there was only one university school of nursing at the National and Kapodistrian University of Athens. Recently, many university nursing schools have started functioning in the country. At present, nurses working in the National Healthcare System rarely graduate from university nursing schools. This exaggerates the already existing problems related to subservient interactions between nurses and physicians.

Considering nursing personnel at the core of healthcare provision, researchers are compelled to further explore the conscientious objection of nursing personnel. When a nurse contributes to a medical procedure that goes against her core values and beliefs (namely, against her conscience), this may result in harm not only of their moral integrity but also of their health, since it may cause to herself negative outcomes such as burnout due to a high level of moral distress.

Importantly, the interactions between nurses and physicians should be coherent and used to conduct good communication in healthcare workplaces. Suboptimally or intimidating relationships between physicians and nursing personnel can bring about a situation that may have a devastating impact on patients. A healthy work environment that promotes patient safety requires good communication and collaboration between physicians and nurses [31, 32].

It has been suggested that nurses have more positive attitudes toward collaboration than physicians [30, 32]. In addition, nurses and physicians are reported to have differing opinions regarding what might constitute an operational definition of effective collaboration [30].

### Suboptimal communication and support at work

In line with the findings of this study, the role of nurse leaders has been highlighted in the literature related to the topic of nurses’ conscientious objection. Nurse team leaders should deal with ethical problems, unite their teams, i.e. address team members’ conflicts, and ‘provide an environment of learning from their mistakes, group reflections, and cultural support’ [33]. They should show mentorship, ethical behavior and commitment to professional values. However, it is important to keep in mind that nurse team leaders experience considerable pressure and should be aware of their position [33]. Therefore, according to the authors, ‘nurse leaders need to further develop the understanding of conflicts of conscience through education, well-written guidelines for conscientious objection in workplaces and engagement in research to uncover underlying barriers to the raising of conscientious objections…’. Lamb and Evans et al. found that ‘support from leadership, regulatory bodies, and policy for nurses’ conscience rights are indicated to address nurses’ conscience issues in practice settings’ [17]. In that connection, the Nursing and Midwifery Council, UK, state “Paragraph 4.4 of the Code states that nurses, midwives and nursing associates who have a conscientious objection must tell colleagues, their manager and the person receiving care that they have a conscientious objection to a particular procedure. They must arrange for a suitably qualified colleague to take over responsibility for that person’s care” [34].

Many participants in this study stressed the factors ‘communication with other healthcare professionals or physicians in the workplace’ and ‘adequate support.’ This is not surprising, provided that contextualized relationships and trust are core elements of healthcare. This is emphasized by Milligan and Jones, who state that ‘dialogue and communication lie at the ethical core of human interactions in healthcare’ [31]. These elements are essential for improving the quality of healthcare services. It is the responsibility of each group of health workers to improve communication at workplaces, which can improve patient safety and quality of care in their healthcare institutions.

Furthermore, bullying in the workplace emerged as a substantial barrier to nurses expressing moral objections. Workplace gossip, criticism or even rejection threatens nurses’ psychological well-being and perhaps their careers [35]. This may occur in addition to the fact that sensitive nurses may experience compassion fatigue, empathetic distress, or moral distress due to themselves being constrained from openly expressing their moral concerns about medical procedures in which they have to participate.

### Missing legal protection against job insecurity

The Greek legal framework relating to nurses’ conscientious objection has already been presented above. While conscientious objection has been included in Greek legislation, there is little guidance to help nurses express their conscientious objection. This is also the case for other countries. Czekajewska et al. very recently stated, ‘while the conscience clause is rarely invoked in Poland, most healthcare professionals declare that the current legal regulations in that sphere are unclear and inaccurate’ [22]. Dobrowolska et al. state, ‘Regulation in the United Kingdom is limited to reproductive health, while in Poland, there are no specific procedures to which nurses can apply an objection’ [20]. The guidance of the use of conscientious objection in nursing has a highly political dimension. Eagen-Torkko and Levi have every right to state, ‘Although guidance for the use of conscientious objection has developed in both nursing and midwifery, changes in the political landscape may be creating a source of conflict between providers and the use of conscientious objection’ [36].

### ‘Futile care’ gives rise to nurses’ conscientious objections

Most of the participants in this study raised ethical concerns related to so-called ‘futile care’. Clinical situations that involve futility care are extremely challenging from an ethical viewpoint and often give rise to health providers’ conscientious objection. Katz put it best in saying, ‘If treatments fail to release our patients from the preoccupation with the illness and do not allow them to pursue their life goals, then perhaps that treatment is futile’ [37]. The definition of futile care depends on many factors [38]. Futile care is an excessively complicated concept [38]. Voultsos et al. state, ‘it is extremely difficult to precisely define medical futility, in part because it can depend on subjective aspects such as the values and preferences of individual patients as well as whether a proposed interventions can actually meet its intended goals’ [39]. The stakeholders involved in a clinical situation (i.e. patient, physicians, nurses, relatives/caregivers) may perceive the concept of medical futility differently. The judgment concerning whether a medical treatment is futile involves evaluative judgments. Moreover, the perceived definition of futile care may differ from nurse to nurse [39].

Nurses who feel impeded in expressing their conscientious objection to providing futile care or feel unable to provide palliative care adequately may feel disempowered and/or experience frustration, thus being led to experience moral distress (in the original/strict sense of the term) [40–43]. Moral distress (in the original/strict sense of the term) occurs when a nurse is constrained in some way from taking an action that she considers morally correct [44]. Importantly, nurses’ moral distress may occur with aggressive or futile at the end of life [45]. Nurses are likely to experience moral distress when they witness medical care that they consider aggressive or futile [45]. Prompahakul states, ‘The most commonly cited clinical causes of moral distress were providing futile care for end-of-life patients [46]. In a similar vein, Nikbakht et al. identified causes of nurses’ moral distress related to ‘respectful end of life care’ and ‘futile care’ [47].

### Nurses experienced mild uncertainty about their ethical concerns

Moral integrity is a moral unity between personal and professional values and responsibilities [2]. Nurses’ sense of responsibility and professional values are essential in nursing and ensure patient-centred and high-quality care [48, 49]. Nurses’ sense of responsibility and professional values are essential to making decisions that are ethically responsible.

Importantly, many of the participants in this study appeared to be deeply convicted about the correctness of their moral judgements. However, while participants were detailing their previous experiences with ethically challenging situations involving futile care, reading between the lines (with the help of field notes), the researchers would say that participants had a strong sense of professional responsibility. This sense of responsibility caused participants to feel some amount of moral uncertainty. This last finding deserves further discussion. Morally challenging situations involve, to a greater or lesser extent, some kind of epistemic, social or normative uncertainty. Addressing (i.e. through education), challenging ethical issues (related to nurses’ moral concerns giving rise to conscientious objections) in the environment of nursing practice is complex [17]. Ethical nursing practice requires morally inclusive environments able to address challenging ethical questions raised by nurses [18]. Lamb, Babenko-Mould et al. state, ‘The need for education across nursing, healthcare disciplines and socio-political sectors is essential to respond to nurses’ ethical concerns giving rise to objections’ [18]. Uncertainty about the patient’s medical condition operates as a barrier to making a proper and fair moral judgment about the refusal to provide further medical treatment. Relevant knowledge, training and experience are required for making ethical decisions.

A supportive ethical climate where nurses discuss and share their experiences with other health providers as moral peers is required to address their ethical concerns in clinical practice. Pesut et al. argue that nurses’ conscientious objection should not be arbitrary, for instance based on convenience or fear. Nurses’ conscientious objection should be based on their in-depth reflection upon their own moral response [50]. Nurses should be instructed to conscientiously object to providing care or participating in care after having reflected upon their moral responsibility. Their objection should not be based on what their patient wills (The Nursing and Midwifery Council, UK, The Code, § 20.7., 2015, updated: 2018) [34]. Nurses who are conscientious objectors should reflect carefully, critically and in a detailed way about their intuitions [50]. A supportive ethical climate is essential for nurses to be able to reflect upon their moral responsibility [33, 51]. Panchuk and Thirsk state that conscientious objection may not be a viable option in rural and remote settings in Canada due to the limitations that may exist in these settings, such as external support or staffing constraints [52].

### Constraint distress

Nurses’ claims are less recognized than those of physicians, which have long been accepted [53]. It is argued that nurses cannot object to giving patients indirect aid, such as patient preparation and aftercare, serving meals to patients who underwent a morally rejected (from the perspective of nurses) medical treatment or typing referral letters [53]. Furthermore, a scoping review conducted by Brown et al. found that ‘nurses who had a conscientious objection reported feeling alone, uncertain, and stigmatized and that their objection felt futile due to a lack of meaningful professional support’ [54]. This finding is in line with the findings of this study. Moreover, it is argued that nurses’ decision to raise a conscientious objection to the provision of a particular service means that other healthcare professionals may be required to assume an additional workload that they may resent [9]. In that regard, Neal and Fovargue claim that ‘the compatibility of CO and healthcare professionalism…depends on an ability to set appropriate limits on CO in practice’ [55]. Given that determining the limits of conscientious objection is complex and vague, nurses’ CO can easily act as structural violence by infringing on the exercise of patients’ rights to health care services. Nonetheless, ‘there is consensus that the right to objection among nurses is an important, acknowledged part of nursing practice’ [20]. Note however that nurses’ conscientious objection has not yet received the recognition it deserves. Lamb and Pesut have every right to argue that emphasizing the relational nature of nursing may cause nurses to become aware of themselves as conscientious professionals [56].

At any rate, nurses who are not allowed to raise their ethical concerns and make conscientious objections may develop moral distress. Compromising nurses’ moral integrity and reducing their autonomy may lead to moral distress [57]. Nurses’ moral distress was initially conceptualized in the strict (and most influential) sense of the term, according to the original definition coined by Jameton [44]. Nurses experience moral distress when they feel ‘disempowered or impeded’ in taking the course of action they consider to be ethically right [40]. Mills and Cortezzo state that moral distress has historically been described as a feeling ‘resulting from poor communication, discrepant values, and paternalistic hierarchy’ [58]. However, as the definition of moral distress has later been broadened, it may include not only situations involving nurses feeling constrained (constraint distress) but also situations involving nurses feeling moral uncertainty (uncertainty distress) [44]. Uncertainty distress may vary between groups of healthcare professionals [44]. While participants in this study appeared to have experienced constraint distress, few of them appeared to have experienced (mild) moral uncertainty. As nurses are not the ultimate decider and given the subservient relationship between nurses and physicians, it is most likely that nurses suffer from constraint distress rather than uncertainty distress.

The intensive care unit is an ethically challenging environment in which nurses are most likely to have strong ethical concerns and make conscientious objections. This deserves much attention. Chiafery et al. state that **‘**nursing ethics huddles to decrease moral distress among nurses in the intensive care unit’ [59].

### Factors shaping participants’ core values

The quality of the education they had received, the way they grew up in their family and religion emerged as substantial factors that contributed towards shaping nurses’ values on which a conscientious objection might be grounded.

Not surprisingly, religious beliefs are pointed out in the literature as factors affecting nurses’ conscience-based unwillingness to participate in care in not only Christian but also Muslim countries [21, 60–62]. Brown et al. state, ‘Nonparticipation was influenced by their (a) previous personal and professional experiences, (b) comfort with death, (c) conceptualization of duty, (d) preferred end-of-life care approaches, (e) faith or spirituality beliefs, (f) self-accountability, (g) consideration of emotional labor, and (h) future emotional impact’ [61]. Velasco Sanz et al. state, ‘Different authors point out that nurses’ perceptions and attitudes towards Euthanasia are conditioned by different factors, such as religion, gender, poor palliative care, legality and the patient’s right to die’ [62].

### Nurses considered their remote contribution as participation that can give rise to conscientious objection

Without determining the degree of participation giving rise to conscientious objection, nurses’ conscientious objection can easily act as structural violence by infringing on the exercise of patients’ rights to health care services. It emerges from a literature review that many scholars have concerns about the proper limits of conscientious refusal to participate in particular healthcare activities [63]. Determining these limits is complex and vague. Many theorists have put great deal of effort into getting the line of distinction between blameworthy and innocent participation in a particular healthcare activity as sharp as possible [63]. While the participants in this study complained of a lack of legal security pertaining to their professional rights and job stability, they avoided making any reference to the degree of proximity to an activity (regarded as morally wrongful), which might give rise to conscientious objection. However, they considered their remote contribution as participation that can give rise to conscientious objection. The degree of remoteness or proximity seems to be determined by the participants themselves.

### Implications for future policies

The themes and subthemes that emerged from this study might encourage initiatives in the health care system. From this perspective, lawmakers and healthcare services administrations should put much effort into facing the difficult task of accommodating the nursing personnel’s right to conscientious objection, namely, strike a balance between safeguarding nurses’ right to conscientious objection and safeguarding patients’ rights to medical services. This would contribute to an overall sense of intact moral integrity in nursing, which in turn may positively affect both nursing personnel’s well-being/health and the quality of patient care. Enabling nurses to express their conscientious objection should be viewed as a matter of public health policy. Hampering nurses’ ability to express a conscience-based refusal to fulfil a legal duty might lead to their reluctant participation. Viable conscientious objections in nursing require ethically sensitive healthcare services that tackle the barriers to nurses making conscientious objections and assist nurses in overcoming them. Furthermore, the findings provide suggestions for changes in the current work culture between physicians, nursing managers or leaders and nursing staff. Moreover, ethical support for NHS healthcare professionals with a conscientious objection should be available in health care settings. In addition, education on ethics in nursing schools and ongoing education across nursing sectors is essential to make nurses able to delve into some complex ethical dilemmas and address their ethical concerns, as appropriate. Importantly, the law should protect nurses who are conscientious objectors from job loss, thus empowering nurses in their professional role. In connection with that, the law should clarify the distinction between morally objectionable and nonobjectionable remote participation in nursing activities. Furthermore, selected nurses should be ensured in healthcare branches where they would not have ethical objections. Moreover, an effective referral system must be established. Finally, healthcare services administration should consider nurses’ core values and beliefs.

### Strengths and limitations of the study

To the best of the authors’ knowledge, the study was the first qualitative inquiry that explored nurses’ conscientious objection related to end-of-life care in Greece. Furthermore, all participants in this study had great previous professional experience caring for terminally ill patients. These are strengths of this study. However, the study has some limitations that need to be taken into account when interpreting the findings. The study involved a relatively small number of participants. Furthermore, the researchers have included only female nurses in this study. The study is focused on female nurses. This may be considered an apparent limitation of this study. Moreover, the findings reflect the perceptions and experiences of nurses working in two large tertiary teaching hospitals of Greece in Thessaloniki (a large urban center). The findings might be different from those of nurses working in different settings (e.g., hospitals in remote rural regions). In addition, the transcripts were not returned to all the participants for checking.

## Conclusion

Eight major themes and seven subthemes emerged from the thematic data analysis. Oppressive behaviors in the workplace and subservient interactions between nurses and physicians, suboptimal communication and inadequate support of nurses, perceived ineffectiveness of nurses’ conscientious objections, missing legal protection against job insecurity, provision of care labeled ‘futile’, nurses’ false knowledge and perceptions on medical situations related to conscientious objections, nurses’ fears of isolation bullying and negative gossip in the workplace and a trivial amount of nurses’ involvement in medical decisions emerged as barriers to nurses raising conscientious objection. Most nurses adopted a strong stance (for different reasons) against providing care that they labelled ‘futile’, with a few nurses having adopted a strong stance (for different reasons) against avoiding or stopping providing care that others labelled ‘futile care’. Furthermore, from data analysis, it emerged that some nurses had false knowledge and perceptions on medical situations related to conscientious objections, some nurses experienced mild uncertainty distress about their ethical concerns, nurses considered their remote contribution as participation that can give rise to conscientious objection, a collective conscientious objection raised by nurses might have increased chances of being effective, and upbringing, childhood experiences, education and religion are factors shaping the nurses’ core values.

The findings of this study might be helpful to inform nurses and nursing managers or leaders as well as healthcare policy makers. The results of this study might contribute to addressing the need for creating ethically sensitive health care services and ensuring nurses’ moral integrity and high quality of patient care.

### Electronic supplementary material

Below is the link to the electronic supplementary material.

Supplementary Material 1

## Data Availability

The identified datasets analyzed during the current study are available from the corresponding author on reasonable request.

## Abbreviations

CO: Conscientious Objection
ICU: Intensive Care Unit
NHS: National Healthcare System
