# Supplementary material for: Perceptions and experiences of female nurses when confronted with expressing a conscientious objection towards end-of-life care in Greece
Source: BMC Nurs. 2023 Oct 10;22:372. doi: 10.1186/s12912-023-01555-8 (PMC10563366; doi:10.1186/s12912-023-01555-8)
Supplement: Supplementary file 1 — Supplementary Material 1 [file 12912_2023_1555_MOESM1_ESM.docx]

**Supplementary File 1**

**Themes and subthemes**

**Oppressive behaviors and interactions in the workplace emerged as barriers to nurses raising conscientious objection**

**Subservient interactions in the workplace emerged as barriers to nurses raising conscientious objection**

Participant N2 said,

*‘…we are not yet fully professionals compared to medical doctors, I mean…. I won’t tell a doctor “Don’t do this doc…”’*

Furthermore, Participant N14 said,

*‘…we are people…of course we will express our opinions. To the extent that we are allowed as nurses. I won’t say and do it at the same time, I don’t mean that. We will discuss it first with the doctors. I think we need to have stronger and louder opinions…this is what I think. It would do good to our profession… We are more like executive bodies.’*

Moreover, Participant N17 said,

*‘I would express my opinion if it could be heard or be accepted, it would affect the ultimate decision on certain cases…’*

In a similar vein, Participant N1 explicitly said,

*‘…they might tell me after all “listen, it is me who decides, not you! !”’*

In the same vein, Participant N6 went through the details of how she interacted with others and said,

*‘If I was asked to do so since it is my duty, I wouldn’t have a choice to decline... we are not capable of such decisions… we follow orders and recommendations… but we do talk about it with one another (the nurses)… sometimes with doctors too, but when acting it is a whole different situation.’*

Similarly, Participant N5 described in a few words the subservient relationship between nurses and physicians:

*‘…we are supposed to be associates with the doctors, but in fact we are below them thus I should follow the physicians’ recommendations... meaning that morality just sweeps away!’*

Finally, Participant N16 said*:*

*‘…only this… more information regarding the association between the two specialties, nurses and doctors, this mentality should not exist… that there are the superiors and then the rest who follow them.’*

**Perceived ineffectiveness of conscientious objections emerged as a barrier to nurses raising them**

Participant N17 detailed,

*‘…I would like to act in such manner, but I would never do it because I do realize that no one would take my opinion into consideration, especially when you have recommendations to follow… I will not be heard… someone should… someone above me…’*

Furthermore, Participant N14 said that her conscientious objection would be considered weak. She emphasized,

*‘I just think that my opinion is not so strong. The doctor makes the call’*

Moreover, participant N1 emphasized that her conscientious objection, if raised, would be devalued in her workplace environment. She said,

*‘I have the right to express myself... and I will... but no one will listen to me... I will not be heard… my opinion doesn’t count, there will be another nurse to execute the order in case I say no, thus I will not say no. In case anyone asks I will answer, but my answer will not be evaluated…’*

Participant N8 said in a few words,

*‘No! If I don’t do it [a certain nursing activity], someone else will…’*

**Fears of isolation bullying and negative gossip in the workplace emerged as barriers to nurses raising conscientious objection**

Participant N5 recalled her experience and said,

*‘It is not a common practice anymore, and if it is, this is happening silently… to be frank with you it is either if you will express this to the doctor who will still not be able to help you… The procedure will take place…’*

Interestingly, she added,

*I have worked for several years at ICUs, this is a separate area where anything may happen, mistakes and right things don’t show, only the nurses are present and thus are aware of what is really going on in there...’*

Similarly, Participant N8 said,

*‘The ICU is always like that regardless the fact that we do not say anything... both nurses and doctors...’*

Participant N6 dared to admit,

*‘We are not fully according to the law… this is off the book... I do this off the record and I say it… I didn’t use to say so but since I am very experienced now I may say it’*

**Suboptimal communication and inadequate support at work emerged as barriers to nurses raising conscientious objection**

Participant N6 described,

*‘…of course... if I tell this to some colleague of mine or some random doctor, these people have different personalities and they may be amenable to discussing or not...’*

Moreover, Participant N13 said,

*‘If I did have more support from people... in terms of our profession…’*

In a similar vein, nurse N11 emphasized that she needed support from supervisors (nursing managers) and said,

*‘the supervisor, for instance, if we did get her support..’*

In the same vein, Participant N9 said,

*‘I am scared... If only I could have more support from my supervisor’*

**Trivial amount of nurses’ involvement in medical decisions emerged as a barrier to nurses raising conscientious objection**

Furthermore, Participant N13 said,

*‘…these are really thin lines, because let us be frank… there is always an issue between nurses and doctors, taking responsibilities, at that point you do realize you are on your own, the ball will always be in your court…’*

**Nurses believe that collective conscientious objection raised by nurses might have increased chances of being effective**

In a similar vein, Participant N14 said,

*‘it would be easier… if it was something in collaboration… So that everyone could participate, from the supervisors to all the colleagues.’*

**Missing legal protection against job insecurity emerged as a barrier to nurses raising conscientious objection**

Participant N8 highlighted the lack of adequate legal protection and said,

*‘I may say right now that no one would stop me from doing so but at this very moment I was told that either I do it or I’m fired, how am I supposed to be reluctant and not follow the order… [I need] a legal framework where no one can tell me what to do and what not to do.’*

In a similar vein, participant N18 declared,

*‘…if we had legal rights, meaning that someone could easily tell me whether I may participate or decline on certain occasions… we are not under any legal protection, this of course under the provision that is based on mentality of the supervisor at this given time…’*

Furthermore, Participant N6 described,

*‘there is no law and we are not under any law thus we may not express our opinion, we just follow orders.’*

While the authors of this study did not formally collect information about the participants’ employment status, the information provided by participants during their interviews indicated that many of them had been working in the private healthcare sector. These participants expressed a more intense fear of job insecurity than participants who had never worked in the private health sector.

Participant N1 declared,

*‘I am working for the public sector; thus, if I abstain, I’m not afraid for my career future…*

In the same vein, Participant N7 emphasized,

*‘Ok... there is difference in terms of public or private sector… in the private sector you don’t get to talk… that is not the case for the public sector… In the private sector you don’t talk to keep your job, in the public sector you talk because no one can fire you’*

Similarly, participant N11 said,

*‘We are in the public sector; thus, the penalties do not include getting fired…’*

Participant N3 called for a better legal framework and said,

*‘…legal protection… there should be clear boundaries… what are our legal limits... like this is the case that we may be fired.’*

One participant considered that her transfer to another workplace (because of her being objector) would not be greatly harmful to her because otherwise there would be enormous conflict with her core values and beliefs. Participant N11 said,

*‘…if I’m transferred to another department and thus my moral dilemma would be huge but didn’t really bother me. I’d rather prefer being ok with my consciousness…s’*

**‘Futile care’ emerged as the main reason behind conscientious objection**

**Most nurses adopted a strong stance (for different reasons) against providing so-called ‘futile care’**

Participant Ν16 reported that she was faced with a difficult dilemma. She detailed,

*‘I never had the chance to say it before but especially in the oncology department what really concerns me is whether 85-year-old patients should or should not undergo chemo…whether it is really worth it to extend their lives when it’s a matter of hours if the patient will or will not survive. Either if the patient is under fluids or drugs meaning if the patient is being transfused with blood or any other method’*

And then she added,

*‘Before going to the nursing school I would agree to support final stage patients… there are many needs in blood stocks, or treatments, drugs, the cost… this might be after all a big trouble for the patient, and it doesn’t make any change actually, they basically exchange quality time at home with being exhausted in a cold hospital’*

Participant N5 described in detail her previous experience working in intensive care units (ICUs) and said,

*‘I used to work in ICUs… We had old people or last stage patients and we kept them alive with overdoes of drugs only because this is what the family would ask us to do... just to keep them alive.’*

Then, she added,

*‘…it is absurd...overdose for a patient just to keep them alive for 2-3 more days.. what’s the point to that? Even when the family just wanted to say goodbye to their loved ones...which I’m ok with this idea…but still to keep a human being on a bed which could useful for another patient in need..’*

A few minutes later, she detailed,

*‘…there was a case where we transfused a big amount of blood which we kept doing it all night just because the patient was an administrator at the clinic that I used to work.. Of course, you do understand that it is absurd for 90 years old patient to have him in ICU for care… but if that person is some doctor’s family, not nurse’s, only doctor’s.’*

**A few nurses adopted a strong stance (for different reasons) against avoiding or stopping the provision of so-called ‘futile care’**

Participant N7 said,

*‘Turn off the drug delivery systems because one is dying… no turn them off so that one can die! No you do it! …. I used to turn them off, I don’t anymore… they can do to me whatever they want…’*

Furthermore, Participant N12 said,

*‘…finally, I have said ‘no’ regarding the ending of medications administration… yes I’ve done that…’*

Participant N1 emphasized,

*‘…this is a personal experience... I am a pro on the feeding of a patient even on the occasions where there is no point at all to do such thing… they announce to you that you have 5 days to live and my mother lasted 3 months, which were essential to me… so why stop feeding her? Hope dies after the last breath... this is what I believe…’*

Then, she added,

*‘I have absolutely no objection on this issue, if have their blood taken for extra tests even if they tell you that in 5 hours the patient will die… it might take 3 days instead of 5 hours and these days are really important for the patient… I may consider this* *time period extremely important, I may use this time period to say all the things I never had the chance before to say... who is the one to label this patient that he/she will die in 10 hours and so there is no need for transfusion?’*

A while later she stated,

*‘I’d say that if you have no empathy or if you are not willing to put yourself in the patient’s shoes you can’t label them or set any rules and guidelines to follow.. this is what I believe.’*

**Nurses experienced mild uncertainty distress about their ethical concerns**

**Some nurses had false knowledge and perceptions of medical situations related to conscientious objections**

Participant N12 declared,

*‘it [a normative framework] would help a lot, I think, if there was a framework in ICUs regarding the limits in terms of assisting a patient… I see people fading away in ICUs and there is no point at all... nor ending to this…’*

In the same vein, Participant N5 brought up organizational problems to support her wish for a regulation setting a clear cut-off point beyond which medical duty is abolished. She said,

*‘there should be limits… legal protection… for instance when you say to the doctor “why do you feed the patient” or “why give the patient so many drugs” or “why don’t I get any support” it is not the doctor who answers but the junior hospital doctor* **[**intern] *on duty who answers all these questions, not the attending physician’*

Furthermore, one participant said that she would not hesitate to decline to provide nutrition support to terminally ill patients on the grounds that such a decision would not be as harmful to the patient as other decisions about their medical treatment, in which she would not prefer to get involved. However, this is an arbitrary perception from both a medical and moral point of view. This perception may be harmful to patients. In addition, this perception shows inadequate education on end-of-life care issues. Participant Ν13 said,

*‘… I have said “no” many times that the issue faced was not risky…for example…when feeding a last stage patient.’*

**Upbringing, childhood experiences, education and religion emerged as factors shaping nurses’ core values**

**Nurses considered their remote contribution as participation that can give rise to conscientious objection**
